# Cannabis Legalization in Canada: Reflections on Public Health and the Governance of Legal Psychoactive Substances

**DOI:** 10.3389/fpubh.2018.00220

**Published:** 2018-08-06

**Authors:** Jean-François Crépault

**Affiliations:** ^1^Communications and Partnerships, Centre for Addiction and Mental Health, Toronto, ON, Canada; ^2^Dalla Lana School of Public Health, University of Toronto, Toronto, ON, Canada

**Keywords:** cannabis, drug policy, public health policy, cannabis legalization, regulation

## Abstract

The Canadian government is “taking a public health approach to legalizing, strictly regulating and restricting access to cannabis.” There is, however, no universally accepted definition of a public health approach to cannabis. This paper presents what such an approach is, and is not, and discusses its applicability to legal psychoactive substances more generally. It critically reflects on the role of the public health sector in the governance of addictive substances and activities, noting its function of “responsibilizing” individuals and coaxing them to self-regulate—and the contradiction involved when other state actors involved in governance are actively inciting consumption of those substances and activities.

## Introduction

On June 19, 2018, the Canadian Parliament passed Bill C-45—the *Cannabis Act*—to legalize and regulate the production, distribution, and consumption of cannabis. At the time of writing, Bill C-45 awaits the formality of Royal Assent. In all likelihood, by the time this piece is published, Canadian adults will be able to legally purchase and consume cannabis for non-medical/recreational purposes.

The language of public health has permeated discussions of the form legalization should take and the specific policies to be implemented. The federal government is, in the words of the Minister of Health, “taking a public health approach to legalizing, strictly regulating and restricting access to cannabis” (1). At the same time, cannabis producers propose to advertise and promote their legal products in order to “protect public health and safety” (2). What is a public health approach, exactly? And what role does the public health sector play? This paper will explore these questions, which are relevant not only in view of cannabis legalization, but because the notion of applying a public health approach to the governance of psychoactive substances appears to be gaining currency in Canada (3).

## What a public health approach is

There is no universally accepted definition of a public health approach to cannabis. However, among Canadian proponents of such an approach, there appears to be consensus on its main principles [see (4–8)]. An important initial premise is the pragmatic acknowledgment that humans have used psychoactive substances for millennia, and that drug use occurs on a spectrum, from beneficial/benign to problematic/harmful. Harm is a multidimensional concept, encompassing health harms (to self, to others) and social harms (criminalization, stigmatization, etc.) (9, 10). Implicit in this distinction is the recognition that the laws and regulations governing a substance can themselves cause harm, independent of its intrinsic (chemical) properties.

In the specific case of cannabis, proponents of a public health approach contend that:

- the illegal status of cannabis causes harm to its users by exposing them to criminalization, which furthermore tends to be arbitrarily and inequitably applied (11); and
- for the average adult user, cannabis is relatively benign, with the health harms^1^ concentrated among a subset of users who use it frequently and/or began using it early in life [see (12)]; and therefore
- society is better served by legalizing cannabis, strictly regulating it, and managing the risks through the health system (13, 14).

Public health approaches are characterized by a primary focus on population-level (as opposed to individual-level) factors and outcomes, utilizing measures that “attempt to control the *determinants* of incidence, to lower the mean level of risk factors, [and] to shift the whole distribution of exposure in a favorable direction” [Rose, cited in (15), p. 239; emphasis added]. Thus, the policies associated with a public health approach to psychoactive substances are aimed at the risk factors for related harm, rather than substance use *per se* (6). They include measures curbing availability (e.g., via permitted retail locations, hours of sale, etc.) and accessibility (through controls on price^2^ as well as advertising and promotion), and regulations on the product itself (e.g., its potency and quality). A public health approach also involves education and health promotion interventions that target activities and groups deemed to be higher-risk, e.g., impaired driving and use by children and youth. Finally, it ensures that evidence-based treatment and harm reduction services are available.^3^

Decades of research from the fields of alcohol and tobacco have yielded strong evidence of the effectiveness of these policies—especially controls on price and other restrictions on availability (10, 16, 17). Researchers have also found that these policies are most effectively implemented when a public entity controls distribution and sales (10, 16, 17). These population-level policies ultimately “aim to hold down use” using “soft control measures which apply across the board without singling out specific users” [(18), p. 347]. Crucially, such policies can only be implemented when a substance is legal, leading to the conclusion that “legalization is a necessary—but not a sufficient—condition for reducing health and social harms associated with cannabis use.”^4^ By freeing people who use cannabis from the threat of criminal sanctions, legalization will reduce social harms; to the extent that accompanying regulation is guided by public health principles, it should reduce health harms as well.

This particular control model is not necessarily a blueprint for public health approaches to all drugs, however. The case for *legalization* of cannabis, as opposed to some form of decriminalization, rests partly on its risk profile, which is favorable relative to most illicit drugs as well as alcohol and tobacco [see (9, 19)]. Indeed, another proposal from some advocates of health-focused drug policy reform is ensuring that the level of control on a substance is proportionate to the level of risk or harm it poses [see (16)]^5^.

**Figure 1:**
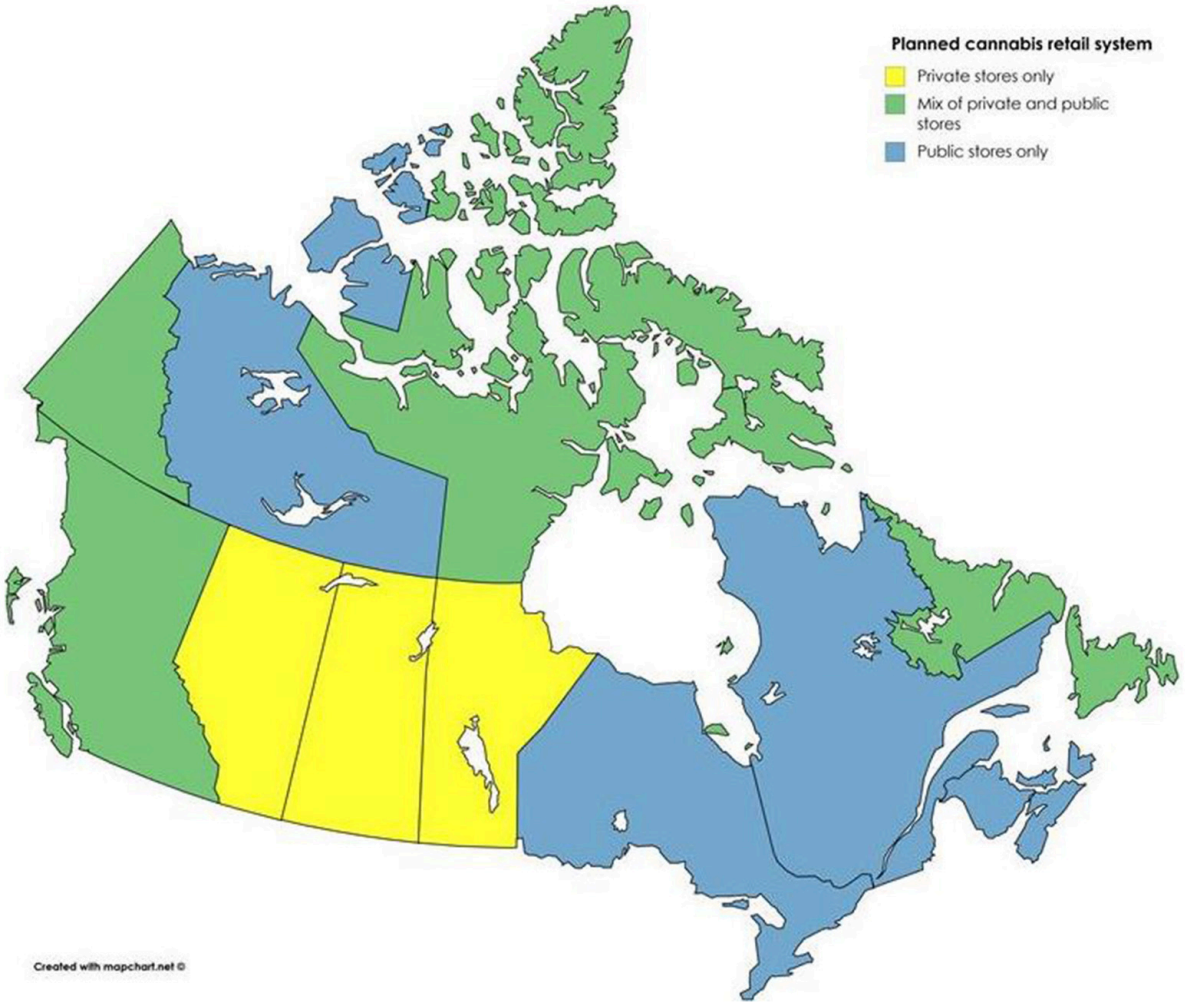
Retail data from (22).

In Canada, senior government officials leading the legalization process have stated that they are committed to implementing a public health approach to cannabis (1, 21). Bill C-45 and its accompanying regulations are for the most part in line with this stated intention, with strong controls on product packaging, advertising, and taxation/price. However, many areas of regulation have been left to the provinces and territories. Most notably, each province and territory will be responsible for determining the legal minimum age (with 18 as the lowest the federal government will allow), how cannabis will be sold within its borders, and where it can and cannot be consumed (e.g., in public, in licensed premises, etc.). Provincial approaches vary greatly [see (22)]. All have opted to harmonize their minimum ages for cannabis and alcohol−18 in Manitoba and Québec, and 19 everywhere else. Some will allow cannabis smoking and vaping wherever tobacco smoking and vaping are allowed, while others will restrict it entirely to private residences. And while some jurisdictions are opting for a retail model in which cannabis distribution is regulated by government but operated by the private sector, others are establishing a public monopoly on sales (see Figure 1)—a model that, as mentioned, is more consistent with a public health approach and more likely to lead to positive health outcomes.

## What a public health approach is not

**Figure 2:**
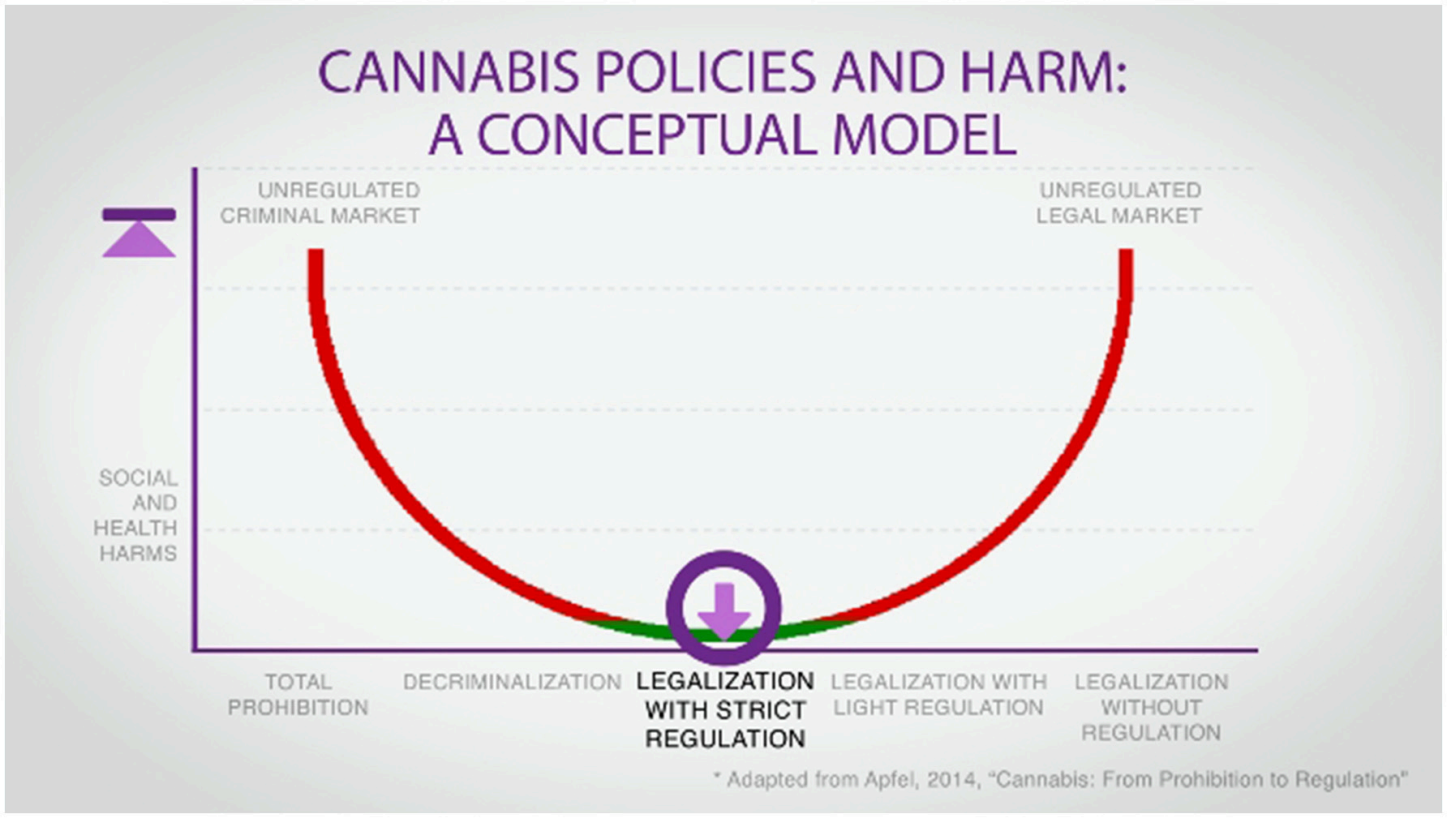
[(5), p. 11].

Drug policy is frequently described as a spectrum, with total prohibition at one pole and an unfettered free market at the other (see Figure 2). Neither extreme is compatible with a public health approach. As Canada moves away from prohibition toward legalization with strict regulation, the social and health harms associated with the former should decrease. However, a new challenge presents itself: ensuring that the new regime does not swing too far in the direction of a commercial system. For on this matter there is consensus in the world of public health: cannabis and the entities producing and selling it should be tightly regulated, with health considerations taking precedence over commercial and fiscal ones at every step (5, 7, 14). The rationale for this position is simple:

- Cannabis use comes with risks, and these risks rise substantially with frequent/heavy use. It is, as often stated of alcohol, “no ordinary commodity” (16).
- Businesses are profit-maximizing entities; in the case of the cannabis industry, the primary and overriding goal is, and will continue to be, maximizing revenues.

Taken individually, neither of these statements is novel or controversial. They are, in fact, rather banal. Yet when considered together, they are in clear contradiction, which from a health perspective can only be reconciled through strict controls on the entities in question. As already mentioned, a public health approach to cannabis *by definition* involves measures designed to hold down consumption levels.

Canada has a rapidly expanding cannabis production industry—a creation, in effect, of the federal government. In 2013, the then-Conservative federal government introduced a number of reforms to Canada's medical cannabis system, one of which was to open cannabis production to the private sector (23). While for-profit production is by no means the only possible model in a legal market,^6^ events since then have essentially negated the alternatives. As early as 2013—almost 2 years before legalization had become a federal election issue—observers were reporting a “green rush” of cannabis investment by investors seeking to “make billions on the legalization of pot” (24, 25). Since then, the number of licensed cannabis producers has gone from just one to over 100; collectively, they have been valued at over $29 billion (26, 27). The purpose of business—indeed, in the famous words of Milton (28), its sole responsibility—is to increase its profits. To expect anything else from the cannabis industry would be naive.

Indeed, although Canada's licensed cannabis producers face strict marketing regulations, particularly around making health claims, they have continually pushed those boundaries (29–31). And while the federal government's Task Force on Cannabis Legalization and Regulation (32) recommended a near-total ban on cannabis advertising and promotion (with the sole exception of the point of sale), and Health Canada (33) recently unveiled packaging guidelines with strict limits on branding, licensed producers have pushed back (34). Ambiguous wording in the restrictions on advertising and promotion in Bill C-45 has encouraged cannabis producers to campaign for the right to advertise and promote. They argue that their proposed advertising guidelines, which would allow television and radio advertising among other forms, “will only promote brand preference, and will not attempt to influence adult non-consumers of psychoactive cannabis products to become consumers” (2)^7^. But the goal of advertising is not only to supply existing consumers but to create new ones, and the evidence is clear that alcohol and tobacco advertising are associated with increased consumption, earlier initiation, and increased harms (36, 37). Emerging evidence suggests that the same is true of medical cannabis advertising (38).

A public health approach is not inherently or necessarily opposed to profit, even in the context of sales of risky products. It does require, however, that business and commercial interests be subordinate to health considerations. Both logic and evidence tell us that an unregulated or lightly regulated cannabis industry would not restrain its efforts to maximize profits. Thus, in the context of a public health approach, the state has the crucial role of “counterbalancing market forces” [(17), p. 96].

## Public health and “responsibilization”

Governance—that is, the processes and practices of governing—is not the exclusive purview of the state. “To the extent that the modern state “rules,” contend Rose and Miller [(39), p. 176], “it does so on the basis of an elaborate network of relations formed amongst the complex of institutions, organizations, and apparatuses that make it up, and between state and nonstate institutions.” Governance is increasingly diffuse, conducted through a multitude of actors and sites.^8^ Correspondingly, the measures involved in a public health approach are implemented and administered by a variety of actors: legislators, regulators, but also medical and allied health professionals, including public health workers, and other experts.

It has also been noted that, in Western societies, governance increasingly occurs not through coercive means, e.g., the use of criminal law, but by enlisting people's own sense of responsibility and encouraging them to govern themselves—essentially, to shape their own conduct (39, 40). As noted, a public health approach to legal psychoactive substances involves measures such as price controls, restrictions on where they can be purchased and consumed, and health promotion campaigns. These interventions are indirect and non-coercive in the sense that they neither directly prohibit individuals from consuming nor punish them if they do^9^. Rather, such governing practices and associated discourses “invite individuals voluntarily to conform to their objectives, to discipline themselves” [(42), p. 11].

As famously observed by sociologists Ulrich Beck and Anthony Giddens, risk has become a pervasive feature of life. Since the 1970s—concurrent with the gradual retrenchment of the welfare state—there has been a shift in discourse around risk and health: whereas under the welfare state it was (at least ostensibly) the state's responsibility to protect the health of its citizens, individuals are now expected to manage their own exposure to health risks (43, 44). This has been referred to as the “responsibilization” of the individual (45). And among the “disciplinary technologies” deployed to responsibilize people—or nudge them into governing themselves—public health features prominently (42, 46). Epidemiological data, for instance, are frequently used to encourage individuals to self-regulate in order to limit their exposure to risk [(47), p. 130]. Through health promotion, public health has been known to frame alcohol or tobacco use as avoidable lifestyle risks—a matter of poor choices, for which the individual is ultimately to blame (42, 48)^10^.

At the same time, however, individuals are encouraged to consume. Our society requires, as all capitalist societies do, healthy, consuming bodies in order to function and flourish (46). Individuals are expected to consume, but to do so responsibly, while “[maintaining] an appropriately responsible attitude toward health” [(48), p. 17]. A paradox emerges: “self-discipline is required to produce commodities, but the consumption of these commodities depends on the gratification of desire, albeit in carefully managed ways” [(42), p. 142]. In Canada, this can be seen in the cases of alcohol and gambling. Both contribute to federal and provincial coffers through taxes; in the case of provinces with public monopolies, substantial revenues are also derived directly, though alcohol sales and gambling losses. Both are heavily promoted by private and public providers. So while public health promotes “disciplined” pleasures characterized by moderation and restraint (50), other actors, including state actors, actively incite consumption^11^.

The gambling sector provides a rich example of these contradictions. Responsibilization of the individual is evident even at the level of language, with programs and policies intended to reduce gambling problems, not only in Canada but globally, referred to as Responsible Gambling. Gambling is widely promoted in Canada as an enticing, pleasurable form of entertainment. Meanwhile, the gambling opportunities on offer in both publicly and privately owned casinos include electronic gaming machines intentionally designed to be “addictive” (52). Individuals are heavily encouraged to gamble, but urged to do so in moderation^12^. The onus to avoid harm is firmly on the individual—not the entities creating, offering, and promoting these risky activities. In the context of a public health approach, even (or especially) where there is a state monopoly, there is certainly the potential for this phenomenon to occur with cannabis.

## Final reflections

This article has suggested that the implementation of a public health approach to cannabis in Canada is a positive development, and that such an approach by definition involves strict regulation of business and active intervention by the state; but also that public health is essentially a site of power, complicit in individualizing health issues and providing cover for governments to benefit fiscally from the consumption of alcohol, gambling—and soon cannabis. In closing we offer three final reflections.

First, it is possible to imagine a government regulating cannabis in a way that considers health and well-being ahead of revenues. Norway, for instance, temporarily banned electronic gaming machines in 2006 due to dramatic increases in gambling problems, and replaced them 3 years later with machines designed to be less problematic—and less profitable (54). In doing so, the Norwegian government sacrificed annual revenues of about $3.5 billion USD^13^. This was possible only because the Norwegian government held a public monopoly on gambling.

Second, if public health is a site of power, this is not inherently negative. It is important that the sector, the individuals in it, and those proposing policies based on its principles, be self-reflexive—“aware of our position as producer and reproducer of certain discourses and practices” [(42), p. 13]. But there does not seem to be any reason that public health *must* be a moral enterprise aiming primarily to instill notions of restraint and risk avoidance in individuals. Cannabis legalization brings with it the possibility of a shift toward focusing instead on countering the commercial interests that would seek to increase cannabis revenues and on addressing the structural factors underlying drug-related harm [see (55)].

Finally, it may be time to consider how a public health approach that includes legalization and regulation might be applied to psychoactive substances beyond cannabis, as a means of reducing the social and health harms associated with the use of those substances^14^. This would be a complex undertaking, with different distribution models required for different classes of drugs—and political and public skepticism all but certain—but these are no reasons to delay the discussion. This is an area where the public health sector is well placed to lead.

## Author contributions

The author confirms being the sole contributor of this work and approved it for publication.

### Conflict of interest statement

The author declares that the research was conducted in the absence of any commercial or financial relationships that could be construed as a potential conflict of interest.

## Footnotes

^1^Health harms associated with cannabis include problems with cognitive, respiratory, and psychomotor functioning, as well as mental health problems and dependence (5).

^2^These can take the form of excise tax, consumption tax, minimum prices, etc. [see (16)].

^3^For a more detailed discussion, see (5).

^4^For a discussion of cannabis *decriminalization*, and why it is inferior to legalization / regulation from a public health perspective, see (12, 13).

^5^This would have implications for alcohol, which is more loosely regulated than cannabis despite being more harmful. Conversely, there are strong arguments for regulating opioids in Canada, in view of the ongoing opioid crisis and, in particular, the contamination of the street opioid supply with fentanyl and various analogs. But given the high level of risk associated with opioid use, such a system would need to be tightly controlled and would bear no resemblance to the distribution models under discussion here [see for instance (20)].

^6^See for instance (8).

^7^For a more detailed discussion, see (35).

^8^This phenomenon has been referred to as “governing at a distance” [(39), p. 173].

^9^There are exceptions, including civil penalties for some offenses and criminal penalties for others, e.g. impaired driving. And though we are referring to interventions around consumption here, it is noteworthy that there are concerns that certain provisions in Bill C-45, especially those dealing with possession and distribution outside of the parameters set by the legislation, “[lay] a new minefield of criminality” [Canadian Civil Liberties Association, cited in (41)].

^10^This sometimes even takes the form of deliberate stigmatization, as in the case of tobacco, where it seems to be a central and intentional part of some health promotion strategies in North America (49).

^11^This concern, in fact, is far from new. In 1932, following the end of alcohol prohibition in Canada and the implementation of a system of government control in eight of nine provinces, a scholar urged readers to consider the “spectacle” of provincial governments “selling millions of dollars' worth of liquors through one department, and engaging men under the education department to persuade youth of the dangers of indulging in those liquors!” [(51), p. 195].

^12^For instance, Ontario Lottery and Gaming has a Responsible Gambling program called PlaySmart, which it bills as offering “Knowledge you can bet on.” PlaySmart offers “information you need to make smart choices and keep the fun in the game” (53). The possibility that the gambling industry or its government overseers may have some responsibility to offer products that are less harmful (52) does not appear to be taken seriously.

^13^Norway likely “saved” money as well through reductions in the direct and indirect costs of problem gambling.

^14^For introductory discussions see (56, 57).
